# Age-Adjusted Charlson Comorbidity Index Scores as Predictor of Survival in Colorectal Cancer Patients Who Underwent Surgical Resection and Chemoradiation

**DOI:** 10.1097/MD.0000000000000431

**Published:** 2015-01-16

**Authors:** Chin-Chia Wu, Ta-Wen Hsu, Chun-Ming Chang, Chia-Hui Yu, Ching-Chih Lee

**Affiliations:** From the Division of Colorectal Surgery (C-CW, T-WH); Division of General Surgery, Department of Surgery (C-MC); Department of Otolaryngology (C-CL); Center for Clinical Epidemiology and Biostatistics (C-HY, C-CL); Department of Education (C-CL); Department of Research (C-HY) Cancer Center (C-CW, T-WH, C-MC, C-CL), Dalin Tzu Chi Hospital, Buddhist Tzu Chi Medical Foundation, Chiayi; School of Medicine (T-WH, C-M C, C-CL), Tzu Chi University, Hualien; and Community Medicine Research Center and Institute of Public Health (C-CL), National Yang-Ming University, Taipei, Taiwan.

## Abstract

We studied the effect of Age-Adjusted Comorbidity Index Score in colorectal cancer patients who underwent similarly aggressive treatment.

Using the National Health Insurance Research Database of Taiwan, we identified 5643 patients with colorectal cancer who underwent surgical resection and chemoradiation from 2007 through 2011. We estimated survival according to Age-Adjusted Comorbidity Index Scores and 5-year survival using Cox proportional hazard regression analysis, adjusting for sex, oxaliplatin-based chemotherapy, socioeconomic status, geographic region, and hospital characteristics.

In the cohort were 3230 patients with colonic cancer and 2413 patients with rectal cancer, who had undergone combined surgical resection and either neoadjuvant or adjuvant chemoradiation. After adjusting for patient characteristics (sex, oxaliplatin-based chemotherapy, socioeconomic status, geographic region, and hospital-characteristics), colonic cancer patients with age-adjusted Charlson (AAC) ≥6 had a 106% greater risk of death within 5 years (adjusted HR = 2.06; 95% CI, 1.66–2.56). In rectal cancer patients, patients with an AAC score of 4–5 had a 28% greater risk of death within 5 years (adjusted HR = 1.28; 95% CI, 1.02–1.61), and those with AAC ≥6 had a 47% greater risk (adjusted HR = 1.47; 95% CI, 1.15–1.90).

Age and burden of comorbidities influence survival of patients with colonic or rectal cancer. Age-Adjusted Comorbidity Score remains an independent prognostic factor even after adjusting for the aggressiveness of treatment.

## INTRODUCTION

Colorectal cancer (CRC) is the third most common cancer and the fourth most common cause of cancer-related death worldwide.^1,2^ In an aging population, the proportion of elderly cancer patients is increasing also.^3^ The incidence of CRC increases with age, as does the prevalence of chronic diseases.^4,5^ Because of improvements in detection and management, more elderly patients with colorectal cancer currently receive adjuvant chemotherapy than they have in the past.^6^

Many CRC patients suffer from one or more comorbidities at the time of their cancer diagnosis,^3^ which may affect screening strategies,^7^ treatment options,^8^ and prognoses.^9,10^ Previous studies demonstrated that CRC patients with coexisting comorbidities, the patients with higher Charlson Comorbidity Index Scores (CCIS), have a poorer survival rate than do those without.^11^ Age-adjusted Charlson (AAC) Comorbidity Index Scores is modified from CCIS after considering age as one additional comorbidity index. The score has been used for survival prediction and treatment options in gynecological and urological cancer treatments.^12,13^ Adjuvant treatment can improve survival even in patients with the highest levels of comorbidity; some patients with comorbidities may forgo chemotherapy unnecessarily, thus increasing avoidable cancer mortality.^14^ Comorbidities and physiologic and functional frailty in aging patients often influence treatment options and outcomes in CRC patients. Less-aggressive treatment in elderly patients decreases the likelihood of receiving adjuvant chemoradiation and leads to poorer prognosis.^15,16^

The combined influence of age and comorbidities on similarly aggressive treatment of CRC patients is still unknown. This study was designed to explore the survival in CRC patients who underwent similarly aggressive treatment of different comorbidity levels adjusted by age. We studied the AAC Comorbidity Index score of CRC patients and the outcomes after both surgical resection and chemoradiation, using the Taiwan National Health Insurance Research Database (NHIRD).

## MATERIALS AND METHODS

### Ethics Statement

This study was approved by the Institutional Review Board of Dalin Tzu Chi Hospital, Buddhist Tzu Chi Medical Foundation, Taiwan. Review board requirements for written informed consent were waived because all personal identifying information was removed from the dataset before analysis.

### Database

Since March 1995, the Taiwan Department of Health has integrated 13 health insurance plans into a universal insurance program. This compulsory social insurance program covers approximately 99% of the residents of Taiwan and has contracts with 97% of medical providers.^17–19^ Taiwan's National Health Insurance (NHI) has the unique characteristics of universal insurance coverage and a single-payer system with the government as a sole insurer. Patients have free access to care with any physician or hospital they choose. The insurance premium is calculated by the insurant's individual monthly income reported to the Bureau. The data for this study were collected from Taiwan's NHIRD for the years 20072011.

**Figure 1:**
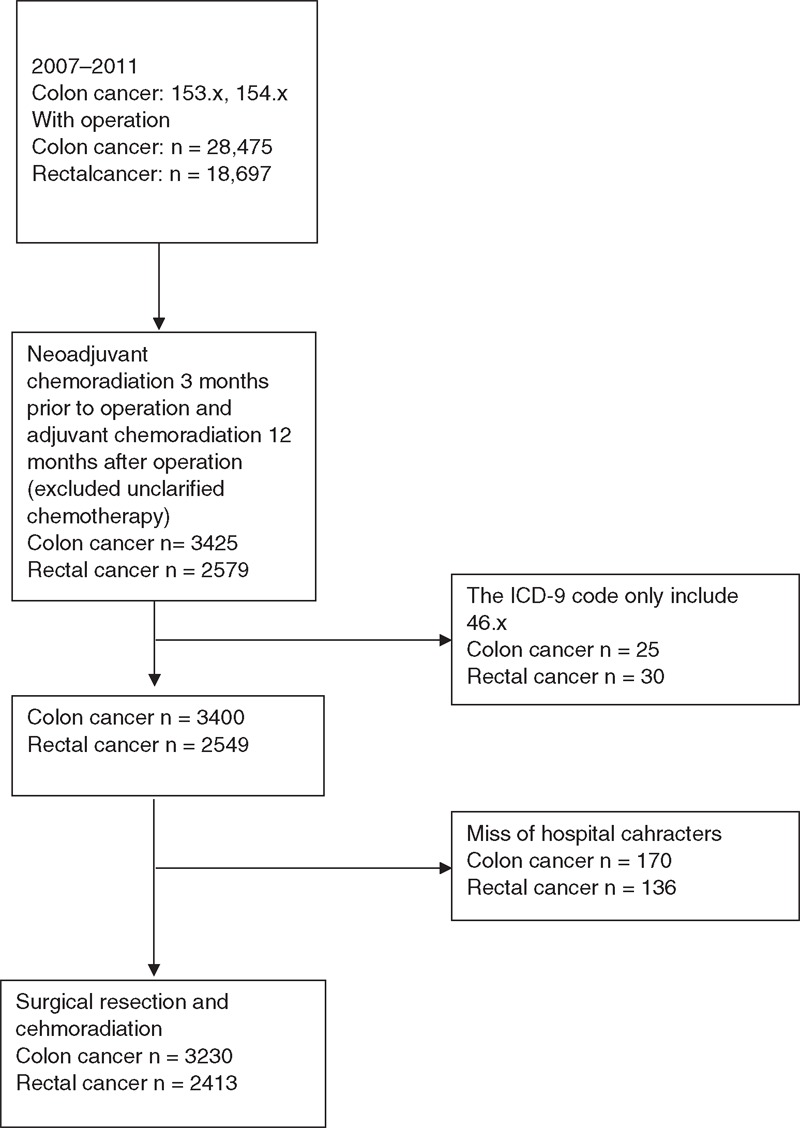
Patient selection flowchart. ICD-9 = International Classification of Disease, Ninth Revision.

Our study cohort consisted of Taiwanese patients diagnosed with colorectal cancer from 2007 to 2011. The patients with CRC (International Classification of Diseases, Ninth Revision, Clinical Modification [ICD-9-CM] codes colon cancer: 153x, rectal cancer: 154x) underwent both surgical resection (45.7x, 45.8, 45.9x, 48.4x, 48.5, 48.6x, 48.74) of colorectal cancer and chemotherapy (oxaliplatin, 5-flurouracil, capecitabine, ufur, or irinotecan) or radiotherapy (92.2x) for their disease during this period. Patients who had chemoradiation both 3 months before surgical resection (neoadjuvant chemoradiation) and 12 months after surgical resection (adjuvant chemoradiation) were included in our study for similar aggressiveness of adjuvant and neoadjuvant treatments. Those patients who received palliative stoma formation (code 46x) only without surgical resection procedures were excluded. A patient selection flow chart is shown in Figure 1.

### Measurement

The key dependent variable of interest was a 5-year overall survival rate after surgical resection. We did not attempt to determine the cause-specific death rate because the registry data we used did not contain this information. The use of overall survival data should not interfere significantly with our results because, as Roohan et al.^20^ have shown in a study adapting a clinical morbidity index for use with ICD-9-CM administrative databases, the survival models for all-cause mortality and cancer-specific mortality do not differ significantly.

The key independent variable of the study was the AAC Index score, which was based on the modified Charlson Comorbidity Index Score (CCIS), a widely accepted measure for risk adjustment in administrative claims data sets.^21,22^ This index is a weighted measure that incorporates age and 19 different medical categories; each is weighted according to its impact on mortality (Table 1). The corresponding ICD-9 codes are listed in Supplement 1. The final score was calculated for each patient by taking into account all comorbid conditions present with the exclusion of colorectal cancer. The age of the diagnosis is adjusted by calculating each decade after 40 years as one point in AAC. For each decade after 40 years, a point is added until 4 points (1 point for age group 41–50, 2 points for age group 51–60, 3 points for 61–70, 4 points for 71 or older). Based on the inpatient and outpatient's codes, the comorbidities recorded from 6 months to diagnosis were calculated as AAC (Table 1). In our study, the AAC was categorized into 4 groups: AAC 0–1, 2–3, 4–5, and ≥6.^10,23^ The survival of each colorectal cancer patient was determined by linking that patient's 2007–2011 mortality data with claims data for the surgical resection up to 5 years before death. Patient characteristics included sex, geographic location, and oxaliplatin-based chemotherapy. Socioeconomic status (SES) presented as the income-related insurance premium was divided into two groups.^24^

**Table 1:**
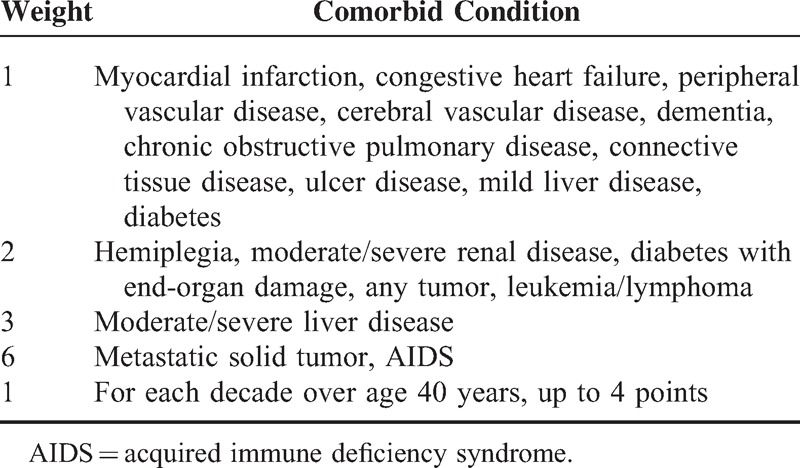
Age-Adjusted Charlson Comorbidity Index

### Other Variables

Hospitals were categorized by hospital accreditation level (medical center, regional hospital, or district hospital) and ownership (profit organization, non-profit organization, and public). The geographic regions were recorded as northern, central, southern, and eastern Taiwan.

### Statistical Analysis

All statistical operations were performed using SPSS (version 15, SPSS Inc, Chicago, IL). Pearson χ^2^ test was used for categorical variables such as sex, age, SES, level of urbanization, geographic region of residence, AAC groups, and hospital characteristics (teaching level, ownership). The mortality rates between different AAC groups were compared by the use of Pearson χ^2^ test. A two-sided *P*-value (*P* < 0.05) was considered significant. Overall survival was estimated with the Kaplan–Meier method and the difference between groups was analyzed by the use of log-rank test. The Cox proportional hazards regression model was used for multivariate analysis with adjust of sex, oxaliplatin-based chemotherapy, SES, geographic region, and hospital characteristics.

## Results

### Demographic Data and Clinical Characteristics

We studied 5643 colorectal cancer patients who underwent aggressive resection and adjuvant chemoradiation. The mean age of patients with colonic cancer (n = 3230) was 60 ± 13 years and the mean age of patients with rectal cancer (n = 2413) was 60 ± 12 years. Among patients with colonic cancer, 24.5% had an AAC score of 4–5, and 9.7% had an AAC score ≥6. Among patients with rectal cancer, 22.3% had an AAC score of 4–5, and 17.0% had an AAC score ≥6. The characteristics of the study population are summarized in Table 2.

**Table 2:**
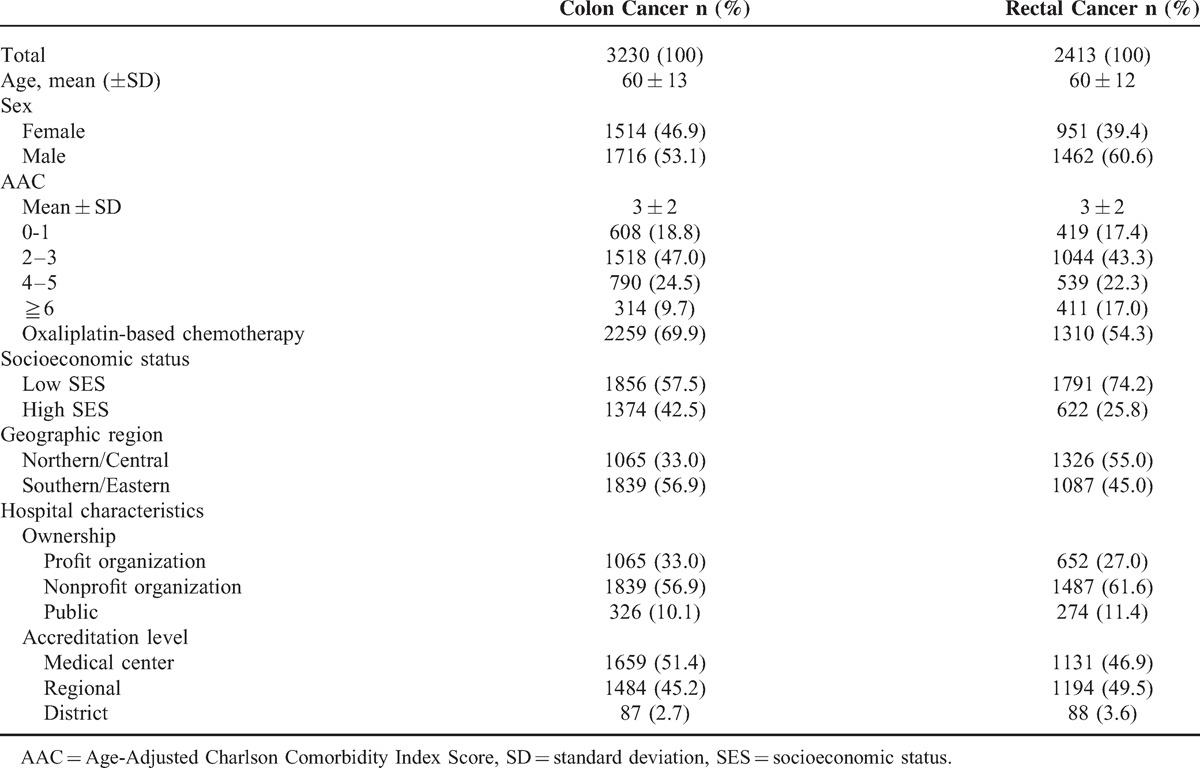
Demographic Characteristics of the Patients Underwent Both Surgical Resection and Chemoradiation From 2007 to 2011

### Univariate Survival Analysis

**Figure 2:**
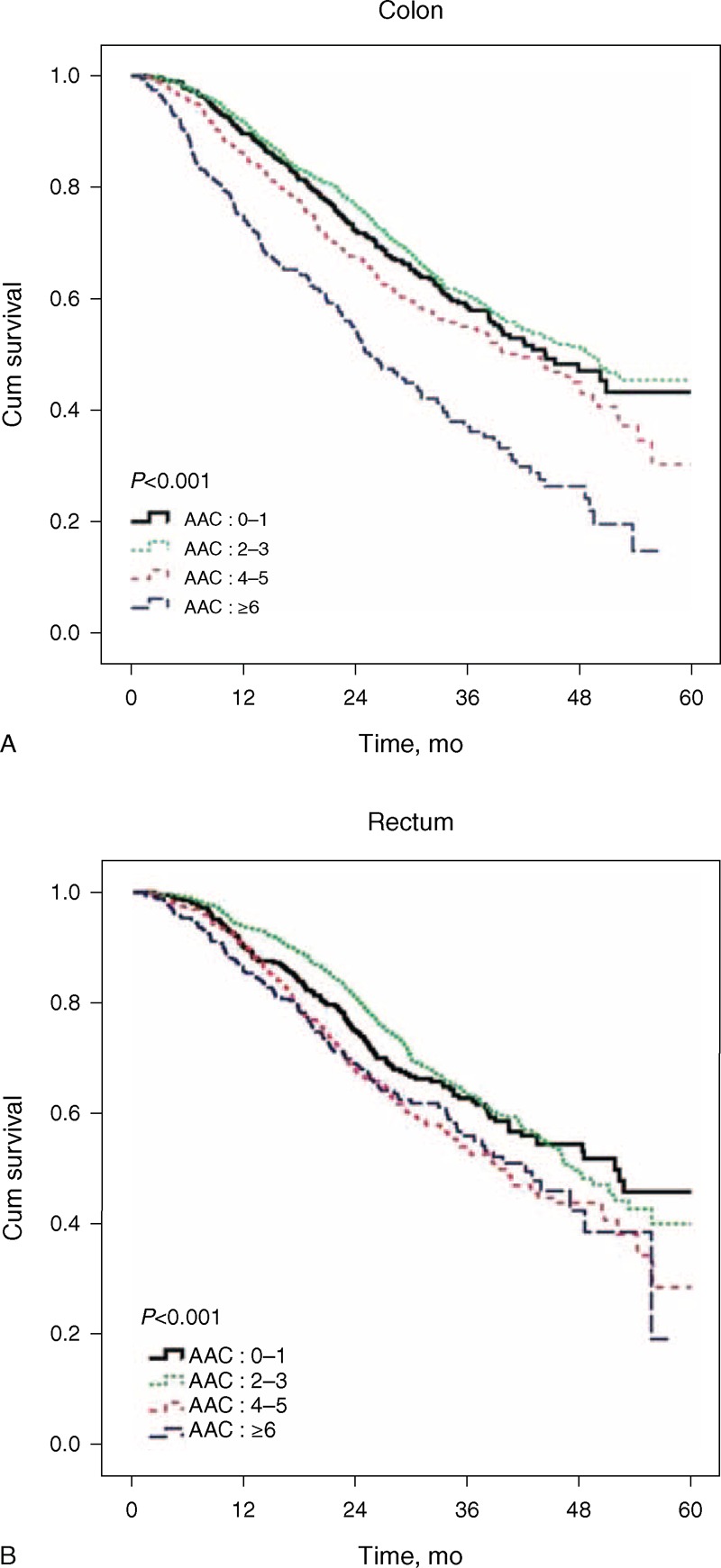
(A) The survival of colonic cancer patients who underwent surgical resection and chemoradiation according to different Age-Adjusted Charlson Comorbidity Index Scores. (B) The survival of rectal cancer patients who underwent surgical resection and chemoradiation according to different Age-Adjusted Charlson Comorbidity Index Scores. AAC = age-adjusted Charlson (AAC).

As can be seen in Table 3, among colorectal cancer patients who underwent surgical resection and chemoradiation, those who had a high AAC score (AAC 4–5 and ≥6) had significantly poorer survival rates than did those with an AAC score of 0–1 (*P* < 0.001). Supplement 2 compares differences in any two AAC categories and also indicates that increased comorbidities significantly relate to poorer outcome. The 5-year survival curves for patients with colonic cancer or rectal cancer are shown in Figure 2A and B, respectively.

**Table 3:**
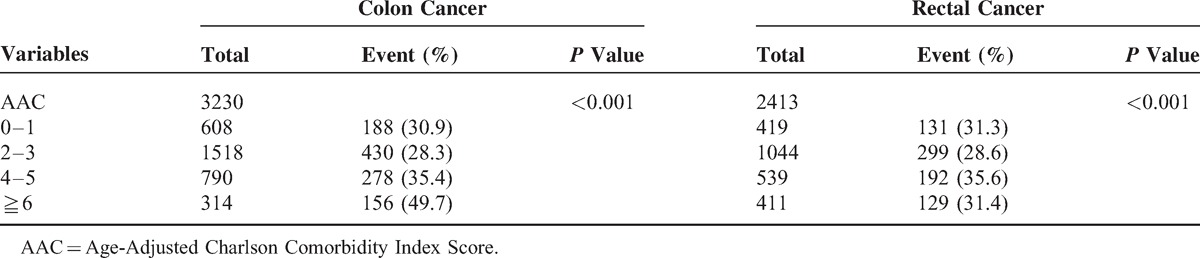
Mortality Among Colorectal Cancer Patients Underwent Resection and Chemoradiation With Different AAC From 2007 to 2011

### Cox Regression Model

In colonic cancer patients with an AAC score ≥6, there was a 106% increase in risk compared with those with an AAC score of 0–1 (adjusted HR: 2.06; 95% CI: 1.66–2.56), as demonstrated with the use of a Cox proportional hazard model. In rectal cancer patients with an AAC score of 4–5 or ≥6, there was a 28% and 47% increase in risk, respectively, compared with those having an AAC score of 0–1 (AAC: 4–5 adjusted HR: 1.28; 95% CI: 1.02–1.61; AAC≥6 adjusted HR: 1.47; 95% CI: 1.15–1.90). The results are shown in Table 4.

**Table 4:**
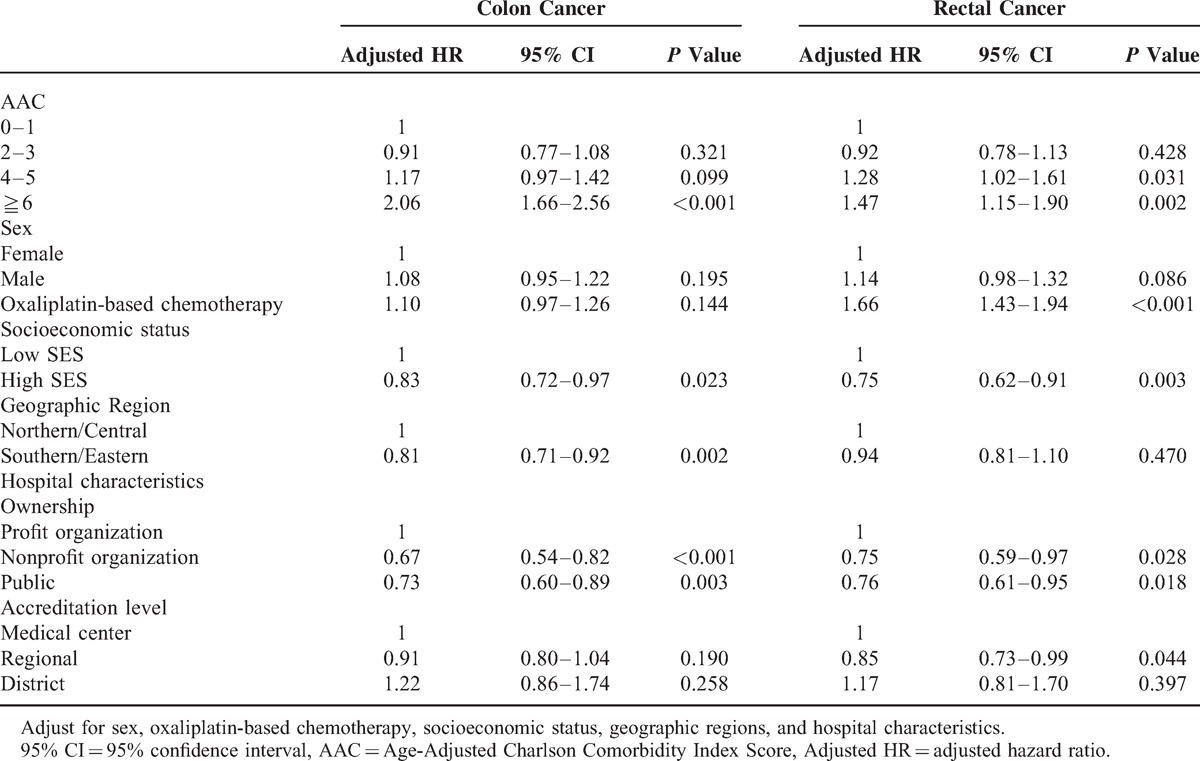
Adjusted HR of Mortality of Colorectal Cancer Patients Underwent Resection and Chemoradiation

## Discussion

Although improved survival has been reported and benefits of chemotherapy have been confirmed in a previous study of CRC patients with multiple comorbidities,^25^ patients with a greater burden of comorbidities are thought to have poor treatment outcome. In this population-based study, patient's age and burden of comorbidities were strong negative prognostic factors after adjusting for sex, oxaliplatin-based chemotherapy, SES, geographic region and hospital characteristics, and even after considering similar aggressiveness of treatment. We studied the patients who underwent similar levels of aggressiveness of treatment in different AAC groups. In patients with both surgical resection and chemoradiation therapy, the AAC score may predict long-term survival and provide a reference for clinical decision-making.

Strengths of this study include its population-based design within NHIRD, and a uniformly organized health insurance system, thus minimizing recall bias and selection bias. Diagnosis of colorectal cancer in our study is valid and definitive. In the NHIRD, biopsy and histological verifications are required before malignancy can be diagnosed definitively as “catastrophic illness” and treatment can be initiated so that the subjects are exempt from copayments for treatment. The diagnosis and quality of cancer information is confirmed based on the NHI Catastrophic Illness card. Neoadjuvant chemoradiation followed by surgical resection, and surgical resection followed by adjuvant therapy, could be identified in NHIRD through ICD-9 codes. Thus, we can analyze treatment outcomes in patients treated with similar levels of aggressiveness but with different levels of comorbidity burden.

We have found similar studies that showed that CRC patients with comorbidities had poorer survival than did those without comorbidities.^14,26–28^ Several factors may contribute to the disparity in survival. First, severe comorbidity is an independent risk factor for death in the cancer patients,^26^ especially in cancer with a low mortality burden, such as localized head and neck cancer and localized colonic cancer.^9^ Second, curative-intent treatment may be compromised by the burden of comorbidity, and thus the aggressiveness of treatment in frail patients may be compromised. Patients with a high comorbidity burden undergo less surgical resection, and CRC patients with comorbidities may receive less adjuvant therapy than do those without.^14,15,25^ Third, there is some interaction between comorbidities and the aggressiveness of cancer.^29^ Medication administered to treat comorbidity may influence the treatment outcome. For example, metformin is associated with a reduction in CRC-related deaths.^30^ The influence of comorbidity on delayed cancer diagnosis is still controversial. In some reports, comorbidities may delay CRC diagnosis by masking symptoms of the cancer,^31,32^ and several conflicting reports have shown that closer medical followup of patients with comorbidities could also lead to detection of colonic cancer at earlier stages.^10,33^

There are several limitations in our study. The Age-Adjusted Charlson Index to measure comorbidity has been tested and shown to be reliable and valid;^34^ however, it does not represent functional impairment, which could influence mortality.^35,36^ Another limitation was our lack of access to detailed information on colorectal cancer stage, histology, pattern of relapse, and other risk factors, such as tobacco use and dietary habits. Although resection of colorectal cancer and chemoradiation involved more-extensive surgery than did palliative diversion, the precise extent of surgery and type of lymph node dissection was not specified. Another limitation is that if the patients received treatment outside of NHI Payment Guidelines (and therefore opted for self-payment), the data would not be shown in NHIRD. Targeted therapy such as cetuximab and bevacizumab was not included in NHI before 2009; however, given the robustness of the evidence, statistical analysis, and sensitivity analysis in this study, these limitations are unlikely to compromise our results.

In conclusion, even among CRC patients who underwent surgical resection and chemoradiation, a higher AAC score is associated with poorer outcome. Although we know that comorbidities influence cancer survival, our findings provide physicians and patients with a reference for decision-making in elderly patients and in those with a greater comorbidity burden. Because of an increasing proportion of elderly patients and patients with comorbid conditions among patients with colorectal cancer, comorbidities increasingly must be considered in an era when aggressive treatment of cancer is common.
